# Potential therapeutics against neurological disorders: Natural products-based drugs

**DOI:** 10.3389/fphar.2022.950457

**Published:** 2022-08-19

**Authors:** Abdur Rauf, Md. Mominur Rahman

**Affiliations:** ^1^ Department of Chemistry, University of Swabi, Swabi, KPK, Pakistan; ^2^ Department of Pharmacy, Faculty of Allied Health Sciences, Daffodil International University, Dhaka, Bangladesh

**Keywords:** neurological disorders, natural products, potential therapeutics, drugs, Malnutrition

## Abstract

Neurodegenerative disorders, which are defined by the breakdown of neurons over time, are affecting an increasing number of people. Stroke, Alzheimer's, Parkinson's, Multiple Sclerosis, Migraine, and Amyotrophic Lateral Sclerosis are just a few examples of brain disorders that have no cure. Besides, there is a huge demand for drugs that can cure the diseases mentioned above because the majority of the medications we use to treat them only alleviate diseases. Different neurological disorders have responded satisfactorily to the pharmacological effects of medicinal plants. Despite the numerous multiple types of plants in the world, only a small number of them have been investigated for neurological disorders. As a result, there are many opportunities in this area for further research on plants and their bioactive chemicals. The search for natural therapeutic alternatives that promote faster healing and adverse effects avoidance has gained popularity in recent years. The aim of this mini-review is to explore some natural products that have strong therapeutic effects on neurodegenerative disorders such as Stroke, Alzheimer’s Disease, Parkinson’s Disease, Multiple Sclerosis, Migraine, Amyotrophic Lateral Sclerosis, and others. We have also shown the safety of natural products to improve their appropriate usage in neurological disorders from recent literature.

## Introduction

The prevalence of neurological disorders is on the rise and poses a significant public health problem. Parkinson’s disease, dementia, neuro infections, epilepsy, neurological diseases linked with malnutrition, pain associated with neurodevelopmental problems such as stroke, headache, multiple sclerosis, and traumatic brain injuries are all examples of neurological disorders. There are around 450 million people in the world who suffer from various types of mental diseases. Approximately 50 million individuals suffer from epilepsy, and this figure is rising every day (Rahman et al., 2022b; Sorboni et al., 2022). Alzheimer’s and other forms of dementia are expected to double in population in the next 20 years. More than 322 million individuals suffer from serious depression at any given time (Clevenger et al., 2017) and this figure is expected to continue rising. Neurological disorders constitute over 6% of the global burden of disease (Ferrari, 2022). Many low- and middle-income nations bear a disproportionate share of this cost. As a result of the multiple pathogenic pathways linked with neurodegenerative diseases, techniques such as neuroprotection, which includes avoiding cell death and restoring function to injured neurons, may be promising for the prevention and treatment of these disorders (Hartman et al., 2020).

Natural products, such as medicinal plants, phytopharmaceuticals, nutraceuticals, vitamins, and nutritional supplements, are being used more frequently around the world for various health conditions. These products are generally considered to be secure. We can have an evidence-based approach to the safety of these products with the use of several research methods, such as clinical safety studies and pharmacovigilance-based investigations. We have acquired additional knowledge about the safety of natural products to improve their appropriate usage in neurological disorders by compiling papers on this topic from recent literature. An essential overview of the knowledge that is available today on these issues were provided by certain systematic reviews and meta-analyses. The use of natural substances in complementary and alternative medicine can lead to the discovery of new medication lead compounds. To combat neurodegenerative diseases, the use of natural substances has recently emerged as a new sector (Bhattacharya et al., 2022).

Both *in vitro* and *in vivo* studies have indicated that the use of natural ingredients can improve pre-clinical models of neurodegenerative diseases. The phytoconstituents like polyphenolic antioxidants that may be found in herbs, nuts, fruits, and vegetables as well as freshwater and marine flora are among the many natural items that can be used to treat various health problems. These phytoconstituents may be able to reduce neurodegeneration and improve cognitive and memory functioning in the brain. Several neurodegenerative diseases, such as Alzheimer’s disease Parkinson’s disease, epilepsy and other degenerative disorders of the nervous system may benefit from their use (Rahman et al., 2020). Researchers have shown that natural products can have widespread neuro-pharmacological effects by suppressing the production of inflammatory mediators or increasing levels of specific cell survival proteins (Rahman et al., 2021). Opioids, galantamine, and anticholinesterases like physostigmine and neostigmine were all isolated from plants as proof of the relevance of plant-derived bioactive substances in the treatment of neurological disease. Seven of the 26 natural medicines licensed in the recent decade were for the treatment of neurological disease, including three for Parkinson’s disease (Karim et al., 2018; Rahman et al., 2022a).

Even just a small number of plants from across the world have been studied for their potential to cure neurological disorders, therefore there are several directions in which this area of study might be expanded. In recent years, there is a growing interest in the quest for alternative treatments based on natural products, offering better recovery and the avoidance of side effects. This special topic was a platform for relevant experts in the field of ethnopharmacology and neuropharmacology to share cutting-edge research and emerging literature-based reviews related to neurological disorders. The primary goal of this study was to examine research and reviews connected to the creation of novel medications derived from plant sources against neurological disorders and created interest in the prevention and treatment of neurodegenerative diseases as well as confirmed the safety of natural products to enhance their proper use.

In conclusion, medicinal plants are a vital source of a wide range of bioactive chemicals. Ethnopharmacology studies can lead to the development of more effective multi-target drugs for therapeutic approaches to various diseases, such as neurological disorders, in addition to providing a scientific basis for the optimal dose and possible toxicological effects on the local community.
